# Accumulated biomechanical effects of mandibular molar mesialization using clear aligners with auxiliary devices: an iterative finite element analysis

**DOI:** 10.1186/s40510-023-00462-7

**Published:** 2023-04-10

**Authors:** Xinwei Lyu, Xing Cao, Luxian Chen, Yuyao Liu, Huilin Li, Cheng Hu, Jiali Tan

**Affiliations:** 1 Department of Orthodontics, Hospital of Stomatology, Sun Yat-Sen University, Guangzhou, 510055 China; 2 Guangdong Provincial Key Laboratory of Stomatology, Sun Yat-sen University, Guangzhou, 510080 China; 3 Guanghua School of Stomatology, Sun Yat-Sen University, Guangzhou, 510080 China

**Keywords:** Finite element analysis, Clear aligners, Numerical simulation, Molar mesialization, Long-term tooth movement, Biomechanics

## Abstract

**Background:**

The biomechanics generated by the clear aligner (CA) material changes continuously during orthodontic tooth movement, but this factor remains unknown during the computer-aid design process and the predictability of molars movement is not as expected. Therefore, the purpose of this study was to propose an iterative finite element method to simulate the long-term biomechanical effects of mandibular molar mesialization (MM) in CA therapy under dual-mechanical systems.

**Methods:**

Three groups including CA alone, CA with a button, and CA with a modified lever arm (MLA) were created. ﻿Material properties of CA were obtained by in vitro mechanical experiments. MM was conducted by the rebound force exerted by CA material and the mesial elastic force (2N, 30° to the occlusal plane) applied to the auxiliary devices. Stress intensity and distribution on periodontal ligament (PDL), attachment, button and MLA, and displacement of the second molar (M2) during the iterations were recorded.

**Results:**

There was a significant difference between the initial and cumulative long-term displacement. Specifically, compared to the beginning, the maximum stress of PDL decreased by 90% on average in the intermediate and final steps. The aligner was the main mechanical system at first, and then, the additional system exerted by the button and MLA dominated gradually. The stress of attachments and auxiliary devices is mainly concentrated on their interfaces with the tooth. Additionally, MLA provided a distal tipping and extrusive moment, which was the only group that manifested a total mesial displacement of the root.

**Conclusions:**

The innovatively designed MLA was more effective in reducing undesigned mesial tipping and rotation of M2 than the traditional button and CA alone, which provided a therapeutic method for MM. The proposed iterative method simulated tooth movement by considering the mechanical characteristic of CA and its long-term mechanical force changes, which will facilitate better movement prediction and minimize the failure rate.

## Background

Clear aligner therapy (CAT) has attracted an increasing number of patients and orthodontists for its comfort, esthetics, and individual customization [1]. Different from the conventional fixed appliance, the orthodontic force exerted by a clear aligner (CA) relies on the rebound force after the deformation of thermoplastic material. A series of personalized thermoplastic aligners are produced through the three-dimensional reconstruction of dental models, and computer-aided design and manufacturing [2]. Then, patients wear each aligner for 7–14 days to move the malposition teeth gradually to the target position in a small range. Therefore, an accurate orthodontic force prediction generated by each aligner toward the target position is essential for the therapeutic effect of CAT.

The present computer-aid design and planning process of orthodontic tooth movement mainly depends on morphological tooth positions and the experience of orthodontists. Firstly, the computer determines the clinical and esthetic tooth target position preliminarily. Then, clinicians make patient-specific treatment planning modifications in the software interface, in which the tooth movement is constrained by the prescribed tooth geometry and occlusal function. This information will be dynamically updated as the tooth moves to guide clinicians during the tooth repositioning [3]. However, the material characteristics of CA increase the complexity of the position, distribution, and strength of the mechanical loading system. In addition, the long-term mechanical changes of CA are not considered in the current computer process. Accordingly, the predictability and precise control of tooth movement are still undesirable, especially for bodily movement [4].

The absence of the first molar is common in clinical practice, as the first molar is considered most prone to dental caries and even premature extraction before 15 years old [5]. The alternative treatments for the missing first molars involve prosthetic restoration with dentures or dental implants, autotransplantation [6], and space closure by orthodontics [7]. In the presence of the second molar (M2) and third molar, closing the edentulous space with orthodontics is an optimal option, which could eliminate the damage to adjacent teeth and dependence on exogenous restoration [8, 9]. Apart from that, it is also necessary for extraction cases with medium or minimum anchorage of molars to move the molars mesially for closing the space. Nonetheless, when reaching the target position of different tooth movements in CAT, the achievement rate of molar mesialization (MM) is low, which is considered as an essential but challenging task [10, 11]. For instance, the mesial inclination and lingual rotation of the tooth often occur during molar protraction [7]. In clinical practice, elastics, buttons, or lever arms are generally used to assist the mesial movement of the molars in fixed orthodontic treatment [7, 12, 13], but no such case has been seen in CAT. The biomechanical system of CA during the process, especially with auxiliary devices, remains unclear.

Three-dimensional finite element analysis (FEA) has been widely performed to analyze the biomechanics of orthodontics [14–16]. FEA models structural units established on their geometry, loading, and boundaries and converts the data into algebraic equations to simulate stress and displacement. This biomechanical-based digital approach is ideal for incorporating into the computer-aid design and planning process of CAT. However, most studies about tooth movement of CA focused on analyzing the initial displacement [4, 15, 16], which could not reflect the actual orthodontic treatment environment. Since the dental crown moves toward the target position and CA material stress attenuates [17], the orthodontic force produced by the aligner gradually decreases during displacement. Thus, it is essential for orthodontists to precisely predict the tooth movement from the successive displacement with considering the change of force system. Only a few studies investigating bone remodeling with the FE method have been performed. Kojima [18] and Hamanaka [19] simulated long-term tooth movement in fixed appliances by a series of successive initial tooth movements. However, in those studies, neither the long-term effect of CA nor the constant interaction between aligners and additional devices force system was considered.

Therefore, the purpose of this study was to simulate the cumulative biomechanical effects of CA based on an iterative FE method and provide a promising therapeutic method for MM. A modified lever arm (MLA) was designed and combined with CA. That is, two mechanical systems were incorporated to improve bodily tooth movement. The long-term orthodontic tooth movements of CA with auxiliary devices for mandibular MM were analyzed and determined for the first time.

## Materials and methods

### Reconstruction of patient-specific geometric models

The mandibular anatomic digital models were reconstructed by combining the images of cone beam computed tomography (CBCT) and optical scanning of a healthy 25-year-old woman with normal dentition and without maxillofacial deformities.

**Fig. 1:**
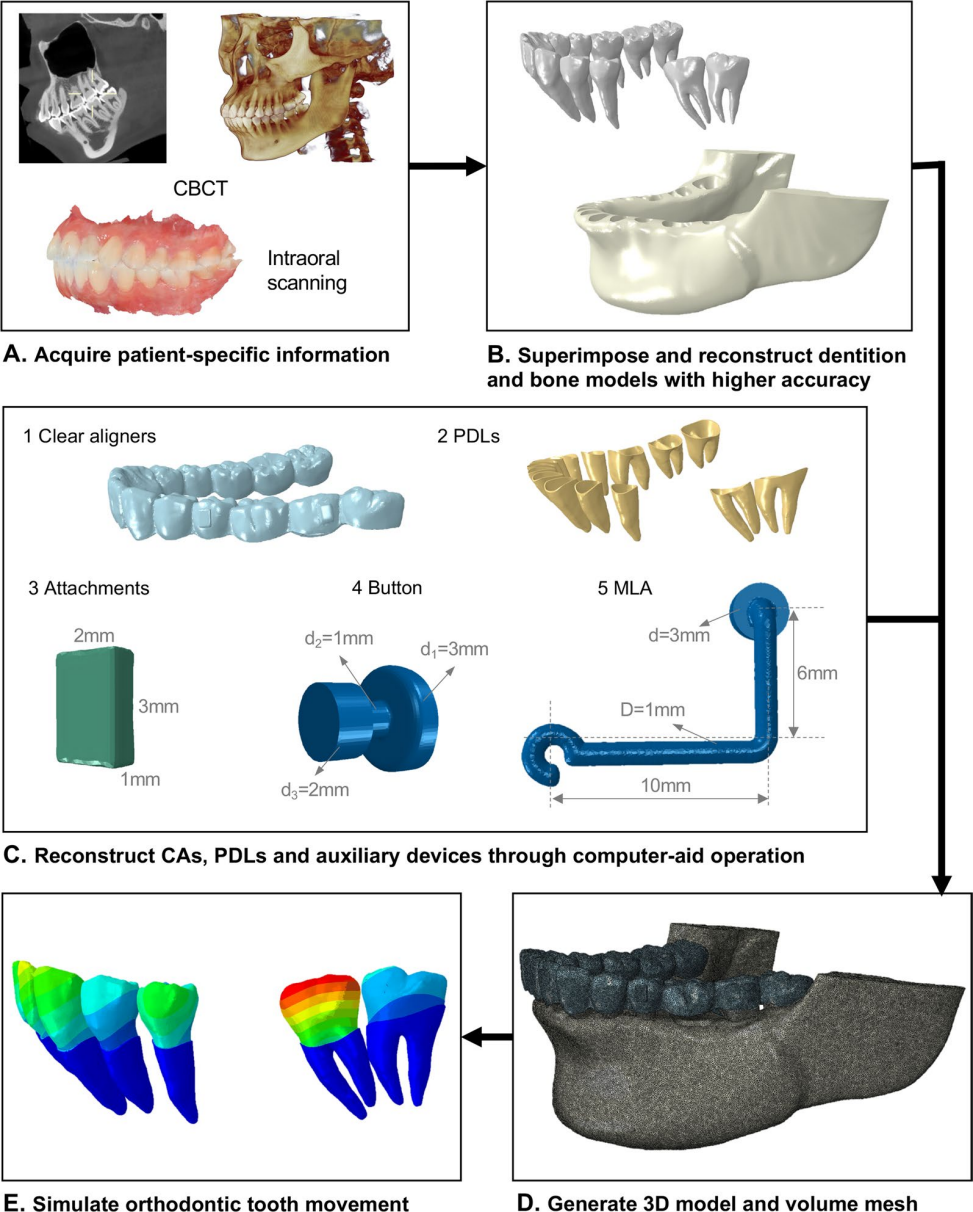
The workflow of reconstructing the FE model

The orthodontic force is transmitted by the contact of the inner surface of CA to the outer surface of tooth crowns, which makes the accurate representation of crowns extremely crucial during biomechanical simulation. Thus, using the oral optical scanner (3shape TRIOS3, Denmark), with a 6.9-μm accuracy, the feature of dental crowns was captured. The CBCT slice images were imported into Mimics 17.0 (Materialise NV, Belgium) and segmented by thresholding to obtain the mandibular bone and individual teeth. Both data from CBCT and the oral optical scanner were fused together with minimum interaction of teeth surfaces in Geomagic Studio 2014 (Geomagic Co, USA). To establish the extraction model, the left mandibular first molar was removed manually. The final digital models with more precise reconstruction were composed of the dental root and mandibular bone obtained by CBCT and the visible surface of the dental crown acquired by optical scanning (Fig. 1A and B).

PDL was simulated as a 0.25-mm-thick uniform layer between tooth roots and bone models [20]. A 0.25-mm-thick shell was added to the surface of tooth roots, and its volume was then subtracted from the alveolar bone to reconstruct PDL.

﻿ Vertical rectangular attachments (3*2*1 mm) were designed on the buccal surfaces of the second premolar and second molar. The button and MLA were virtually modeled using a reverse engineering technique, as shown in Fig. 1C. The mesial end of the cantilever was formed in a semicircular shape to be applied with elastics or hooked over the head of the mini-implants.

The CA thickness was assumed to be 0.75 mm, in which an external offset for the tooth crowns and attachments was developed in the simulation [21]. Through the Boolean operation, the exposed tooth crowns and attachments are subtracted from the thickened crown model to obtain the CA model.

### Material properties

Only the left side of the dentition was used because of its bilateral symmetry. Since the elastic modulus of alveolar bone is much larger than that of the PDL, the orthodontic force will cause only a small amount of bone deformation each step, which is negligible. Hence, the outer boundary of PDL was regarded as a rigid surface, and the alveolar bone was excluded from the FE model [18, 22].

All of the above models were imported to HyperMesh 14.0 (Altair, Troy, Mich) and converted into FE models. The model meshing was constructed with four tetrahedral elements (Fig. 1D). The mesh size was set at 0.10 mm for button and MLA, 0.15 mm for attachments, and 0.20 mm for dentition, PDLs, and CAs, which was more sophisticated than previous studies [4, 23]. The total number of nodes and elements of the three configurations was shown in Table 1. The elements were examined with Mesh Verify command in ABAQUS 2020 (SIMULIA, Providence, RI) to ensure convergence of the FE model.

**Table 1:** Number of nodes and elements of the FE models

Configuration	Total no. of elements	﻿Total no. of nodes
CA alone group	1,626,206	361,110
With button group	1,655,145	367,744
With MLA group	1,681,392	374,464

The mechanical properties of the teeth, attachments, button, and MLA were assumed to be homogeneous, isotropic, with linear elasticity, and defined according to previous studies [24, 25], as shown in Table 2.

**Table 2:** Material properties of ﻿the components in the FE models

Components	Elastic modulus (MPa)	Poisson ratio
Teeth	19,600	0.30
Attachment	12,500	0.36
Button	200,000	0.30
MLA	200,000	0.30

PDL was considered as a hyperelastic–viscoelastic material. A normalized-based relaxation function was adopted to describe the material properties of PDL, and G(I), K(I), and TAU(I) were the parameters in Prony form (Table 3)[26].

**Table 3:** Parameters of normalized-based relaxation function of PDL

	G(I)	K(I)	TAU(I)
1	0.027697	0	0.13927
2	0.097697	0	10.419

**Fig. 2:**
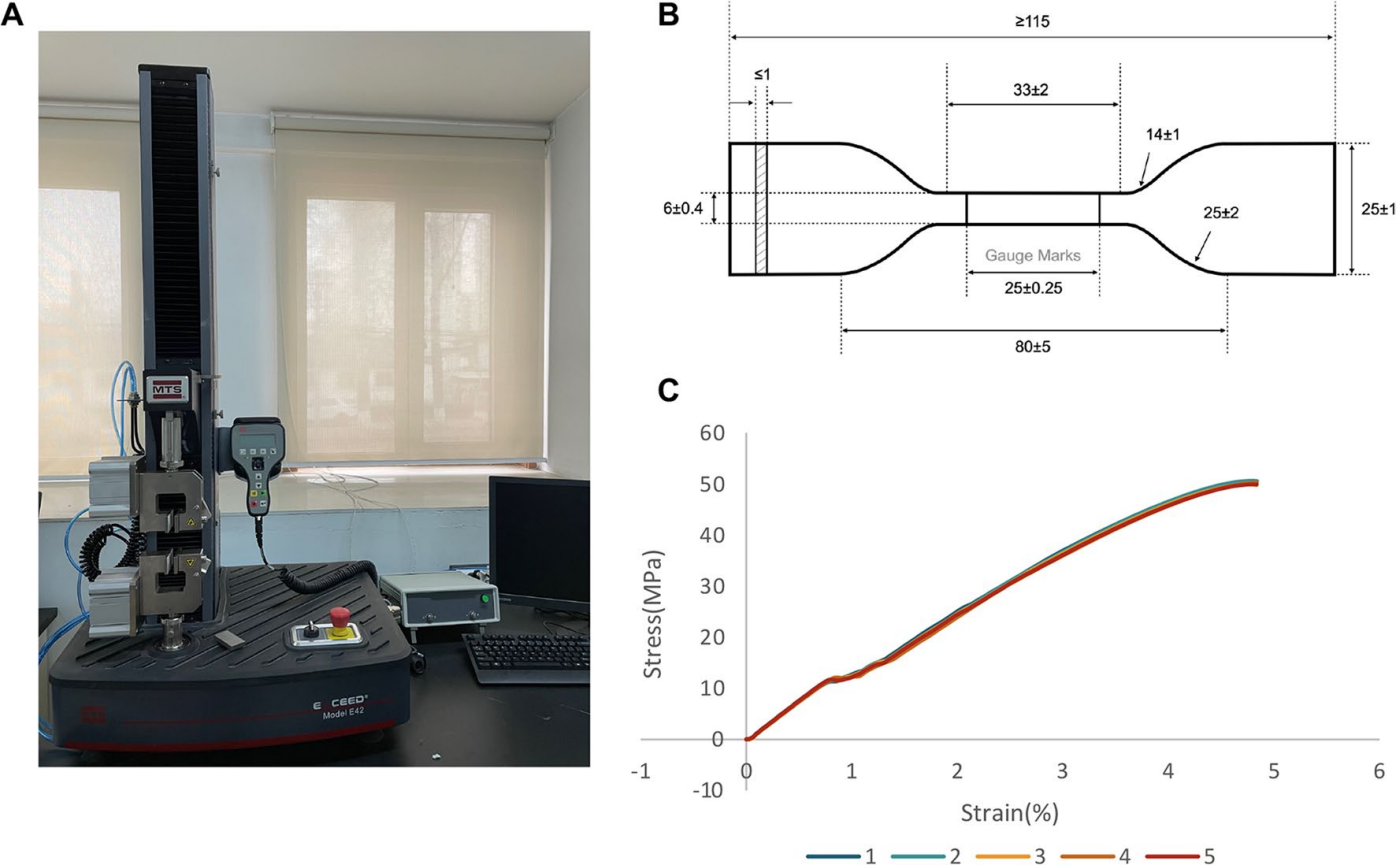
Obtention of CA material parameters by in vitro experiment. **A** Mechanical universal testing machine. **B** Schematic of type A2 test specimen (dimensions in millimeters). **C** Stress–strain curve for nonlinear material properties of 0.75-mm-thick material

CA was assumed to be a nonlinear isotropic elastic and homogeneous material. As shown in Fig. 2, in vitro tensile testing was performed for 0.75-mm thermoplastic sheets (Duran CA®, SCHEU-DENTAL, Iserlohn, Germany), using a mechanical universal testing machine (MTS E42.503, MTS Systems Corp., USA). According to the ISO Norms 527, specimen type 2 cut from five sheets was conducted, and the material properties of CA were determined from the obtained stress–strain curve for further FE analysis.

### Boundary conditions and loading methods

The outer surface of PDLs was set as the fixed boundary region to restrain the model when the force was loaded. The tooth–PDL, tooth–attachments, tooth–button, and tooth–MLA interfaces were assigned “tie” contact interaction conditions. Surface-to-surface contact was assigned to the CA, teeth, and attachments with a Coulomb friction coefficient of *μ* = 0.2 [4].

**Fig. 3:**
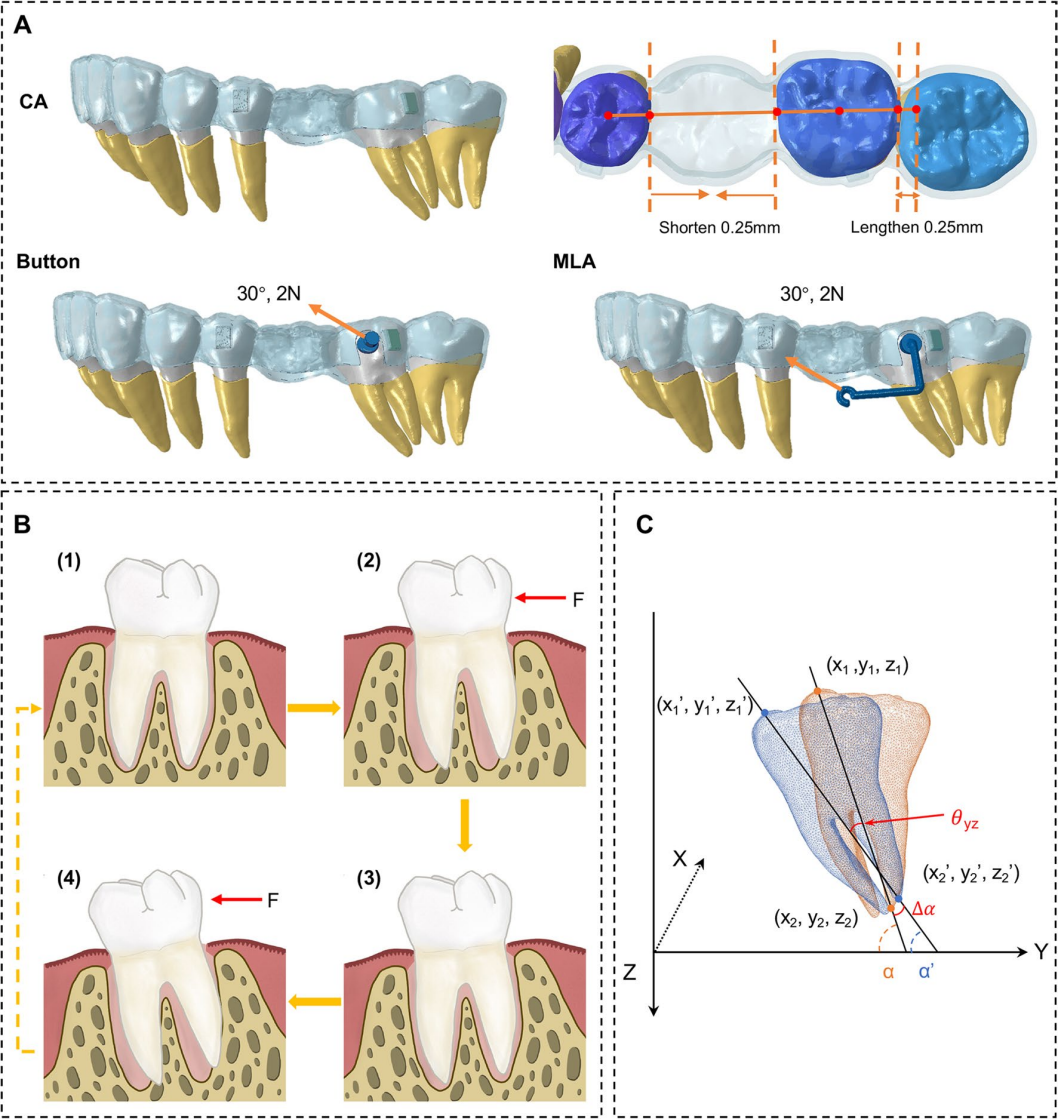
**A** Three configuration groups. **B** Procedure for simulating the long-term tooth movement. The tooth moves by iteration from (1) to (4). (1) The original reconstructed dental, PDL, alveolar bone, and auxiliary devices (not shown). (2) Initial displacement of the teeth and auxiliary devices is calculated. (3) Periodontium remodeling: deformed PDLs and alveolar sockets are updated according to the initial movement of the teeth and are restored to their original configuration. (4) Next step of displacement.** C** Diagram for the rotation angle of M2 in the YZ-plane

Three configuration sets were designed in this study (Fig. 3A).

Configuration 1 (C1): M2 moved bodily toward the mesial direction of the dental arch by 0.25 mm with CA.

Configuration 2 (C2): 0.25-mm mesialization of M2 with CA and a force 2N was applied to the button at + 30° to the occlusal plane.

Configuration 3 (C3): 0.25-mm mesialization of M2 with CA and a force 2N was applied to the mesial end of MLA at + 30° to the occlusal plane.

Figure 3B displayed the simulation procedure for the long-term orthodontic tooth movement, which consisted of two steps: the initial displacement and periodontium remodeling. First, the orthodontic forces from CA and auxiliary devices were applied to the dentition. Then, the initial movements of the teeth and the deformed PDL were calculated. In the second step, the deformed PDL was restored to its original condition with 0.25 mm thickness, and its position was updated according to the initial displacement. The reposition of PDL was based on the assumption that the accumulated long-term tooth movement is correlated with the initial displacement, which has been widely recognized in orthodontic mechanics [27, 28]. The displaced teeth and updated PDL of the last step were assumed to be moved into a stable position and were set as the initial state for the next simulation. Then, these two steps were iterated to simulate orthodontic tooth movement after the long-term process of absorption and apposition of the alveolar bone.

The coordinate x-axis was located in the coronal direction, the y-axis in the sagittal direction, and the z-axis in the vertical direction, with the positive direction pointed to the lingual, posterior, and down, respectively.

The displacement tendency of M2 and equivalent stress and stress distribution of PDL were analyzed. The mean values of four dental cusps (mesio/distobuccal/lingual) and two root apexes (mesial/ distal) points of M2 were measured to represent crown and root displacement.

The difference between the crown and root displacement in the Y-axis was introduced as a reflection of sagittal tipping tendency, as shown in Eq. (1).

1 $$\left( {C - R} \right)_{n} = \mathop \sum \limits_{i = 1}^{n} \left( {c - r} \right)_{i}$$

where (*C*–*R*) represents the total crown–root displacement difference between the initial stage and the *n*th iteration and (*c*–*r*) represents the crown–root displacement difference between the *n-1*th and *n*th iteration.

The rotation angles $\theta$ of M2 in the three planes (XY-, YZ-, and XZ-planes) can be expressed in Eq. (2). The YZ-plane was taken as an example, shown in Fig. 3C.

2 $$\theta_{{{\text{yz}}}} = \frac{180^\circ }{\pi }*\Delta \alpha$$

where $\Delta \alpha$ represents the tooth rotation before and after tooth movement, in radians, which can be obtained through Eq. (3).

3 $$\Delta \alpha = \alpha \prime - \alpha = \arctan \left( {\frac{{z_{2}^{\prime } - z_{1}^{\prime } }}{{y_{2}^{\prime } - y_{1}^{\prime } }}} \right) - \arctan \left( {\frac{{z_{2} - z_{1} }}{{y_{2} - y_{1} }}} \right)$$

The radian between the tooth and coordinate axes before and after tooth movement was denoted as $\alpha$ and ${\alpha }^{^{\prime}}$, respectively.

## Results

### Stress distribution of PDL

The PDL von Mises stress values of each iteration in three groups are listed in Table 4. The maximum PDL von Mises stress was found to be 4.277e + 05 Pa in the first step of the CA group, followed by 4.123E + 05 Pa for the button group, and 3.747E + 05 Pa for the MLA group. The average stress of iteration was the highest for the MLA group (4.921E + 04 Pa) and the lowest for the CA group (4.586E + 04 Pa). The average stress in CA, button, and MLA groups was 10.72%, 11.38%, and 13.13% of the value of the first step, respectively. The stress decreased from the 1st to 15th step in the CA group, while in the button and MLA groups, it reduced first and then stabilized at steps 9 and 5, respectively.

**Table 4:** Maximum von Mises stress of PDL in M2 for each iteration (Pa)

Iteration step	CA	Button	MLA
1	4.277E + 05	4.123E + 05	3.747E + 05
2	1.004E + 05	9.795E + 04	9.231E + 04
3	5.160E + 04	5.744E + 04	6.100E + 04
4	2.689E + 04	3.519E + 04	5.336E + 04
5	2.534E + 04	2.457E + 04	1.602E + 04
6	1.227E + 04	1.518E + 04	1.494E + 04
7	1.010E + 04	8.892E + 03	1.535E + 04
8	6.911E + 03	9.313E + 03	1.391E + 04
9	6.625E + 03	6.400E + 03	1.948E + 04
10	4.049E + 03	5.993E + 03	1.287E + 04
11	3.726E + 03	6.124E + 03	1.262E + 04
12	3.484E + 03	6.146E + 03	1.478E + 04
13	3.207E + 03	5.638E + 03	1.197E + 04
14	2.928E + 03	6.371E + 03	1.179E + 04
15	2.747E + 03	6.585E + 03	1.308E + 04
Mean	4.586E + 04	4.694E + 04	4.921E + 04

**Fig. 4:**
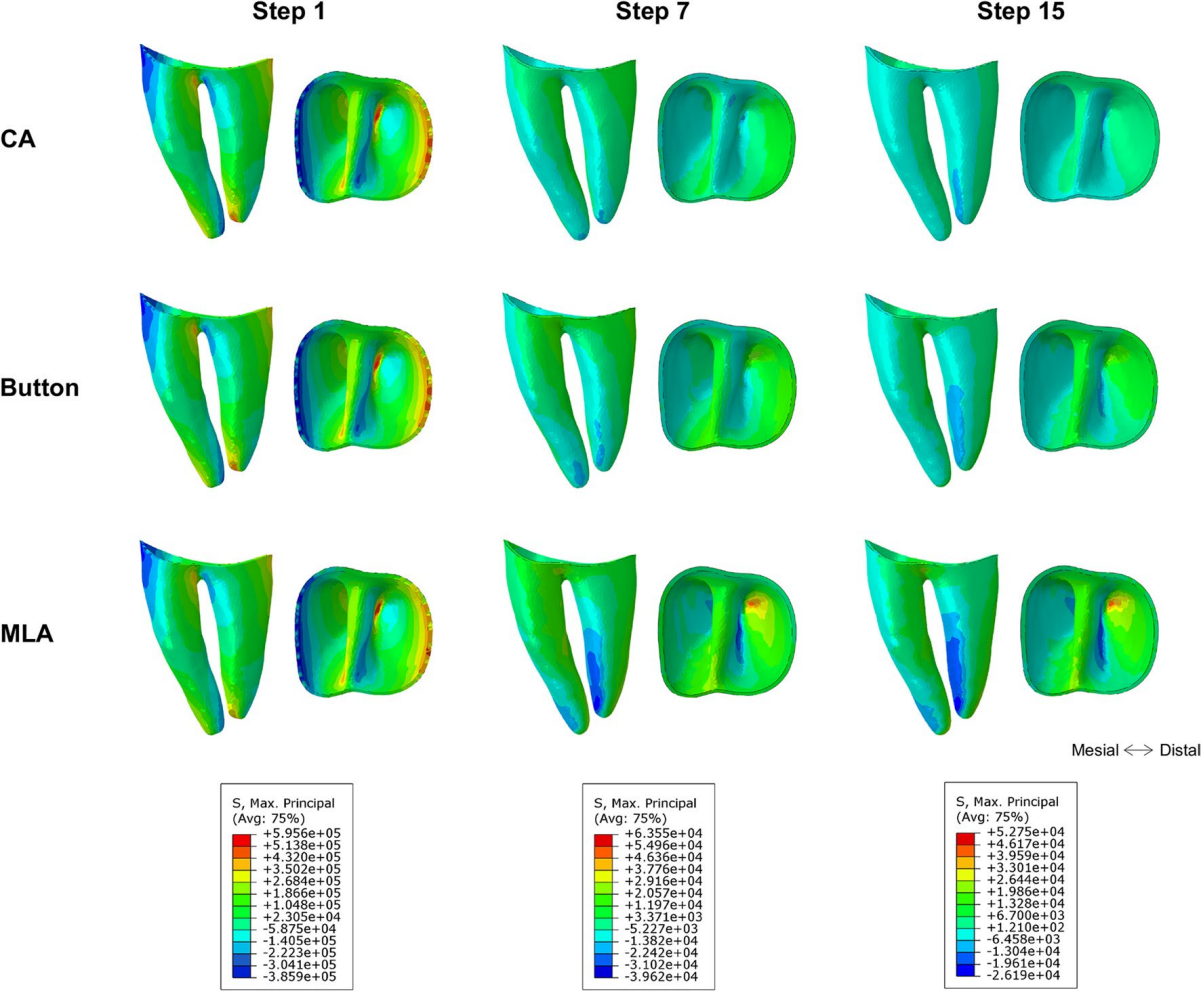
Max principal stress distribution of PDL at the beginning, intermediate, and final steps

Figure 4 illustrated the max principal stress distribution at the beginning, intermediate, and final steps. Stress distribution in the three cases was similar in the 1st step. Compressive stress was observed at the mesial cervix and distal apex, and tensile stress was mainly concentrated on the distal cervix and mesial apex. In the 7th step, stress values decreased in the CA group, and higher compressive stress was transferred to the buccal side of apex, while tensile stress was still in the distal cervix. In the button group, compressive and tensile stress was observed at the buccal and lingual side of apex, respectively. In the MLA group, compressive stress was mainly in the mesiobuccal side of apex, while tensile stress was in the distolingual side of apex, and the stress around the cervix was minor. In the 15th step, CA group, compress stress was observed on the mesial side of PDL, while tensile stress on the distal side. Stress distribution in the button and MLA groups was similar to that in the MLA group at step 7. Compared to the beginning, the maximum von Mises values decreased by 90% on average in the intermediate and final steps.

### Stress distribution of the attachment and auxiliary devices

**Fig. 5:**
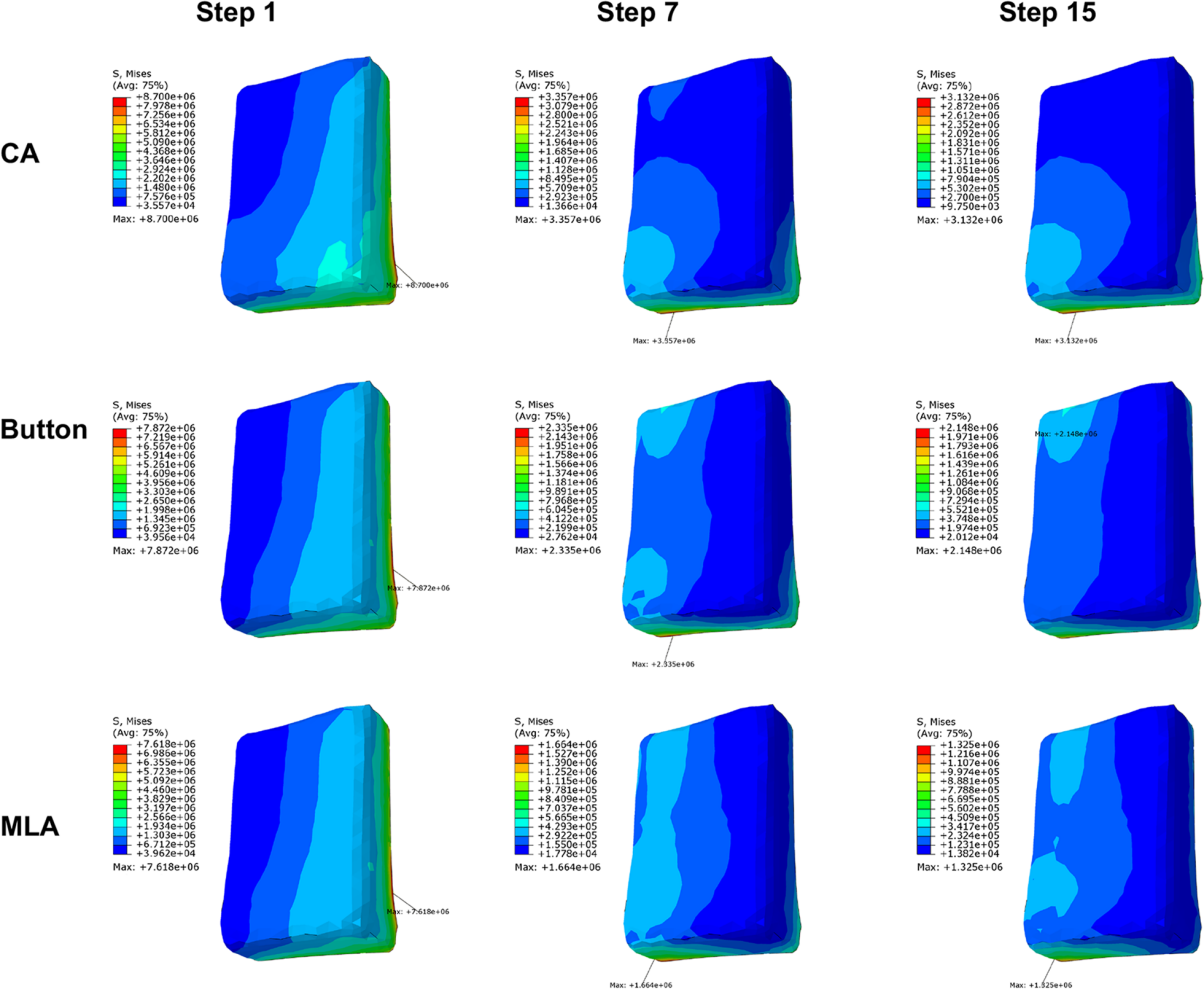
Von Mises stress (Pa) of attachments at the beginning, intermediate, and final steps

The maximum von Mises stress was mainly concentrated on the interface between the attachment and the tooth surface (Fig. 5). With the increase of steps, the stress on the attachment decreased gradually, with higher stress concentration altered from the distal to the mesial position. The intensities of the stress of the attachment were relatively smaller with the addition of the button and MLA.

**Fig. 6:**
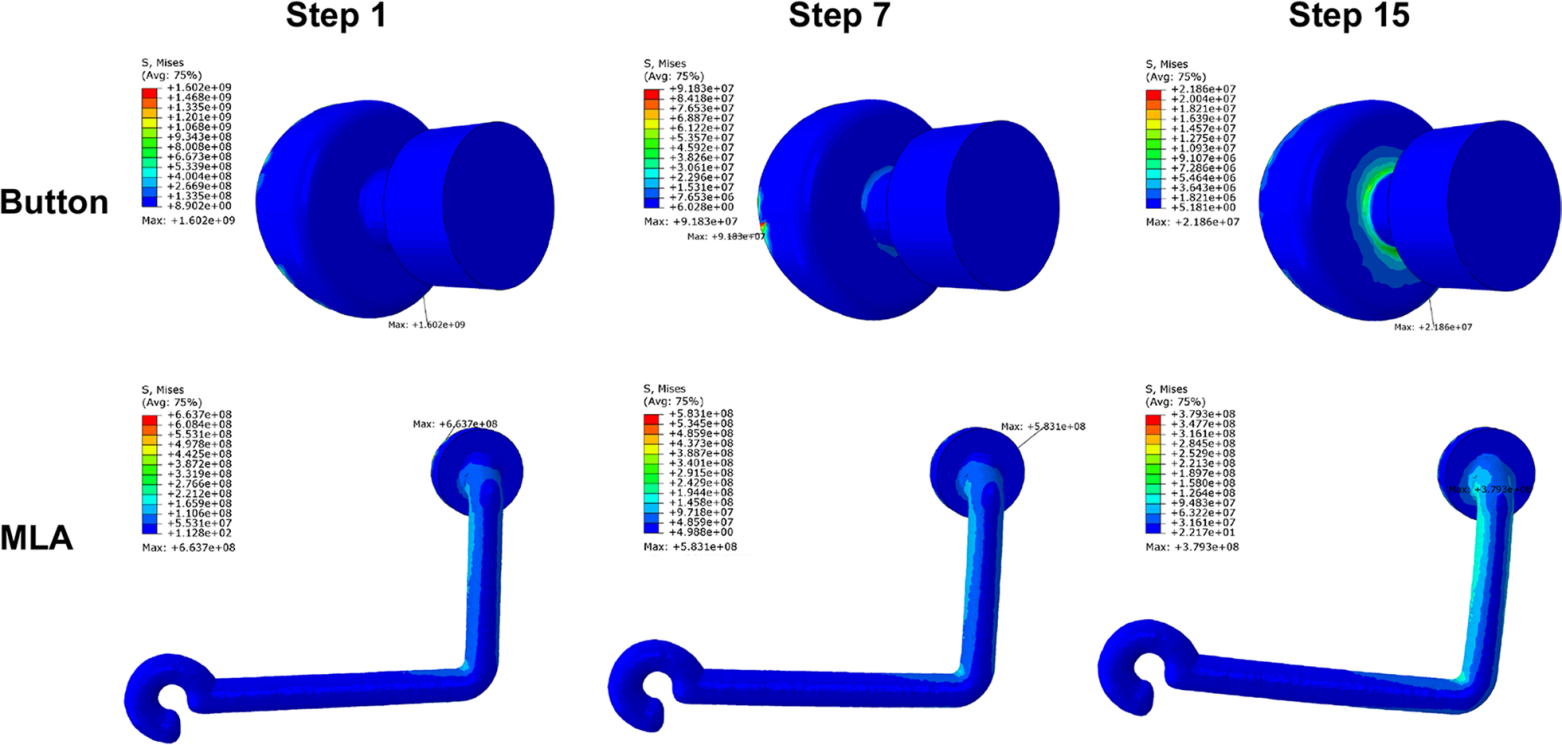
Von Mises stress (Pa) of button and MLA at the beginning, intermediate, and final steps

Button and MLA followed a similar trend (Fig. 6). The maximum stress was noted to be 1.602E + 09 Pa for the button at the beginning and decreased to 2.186E + 07 Pa at the final step, with a reduction of 98.6%. A slightly higher concentration was shown in the middle third of the button and MLA in the 15th step.

### Displacement tendency of the tooth

**Fig. 7:**
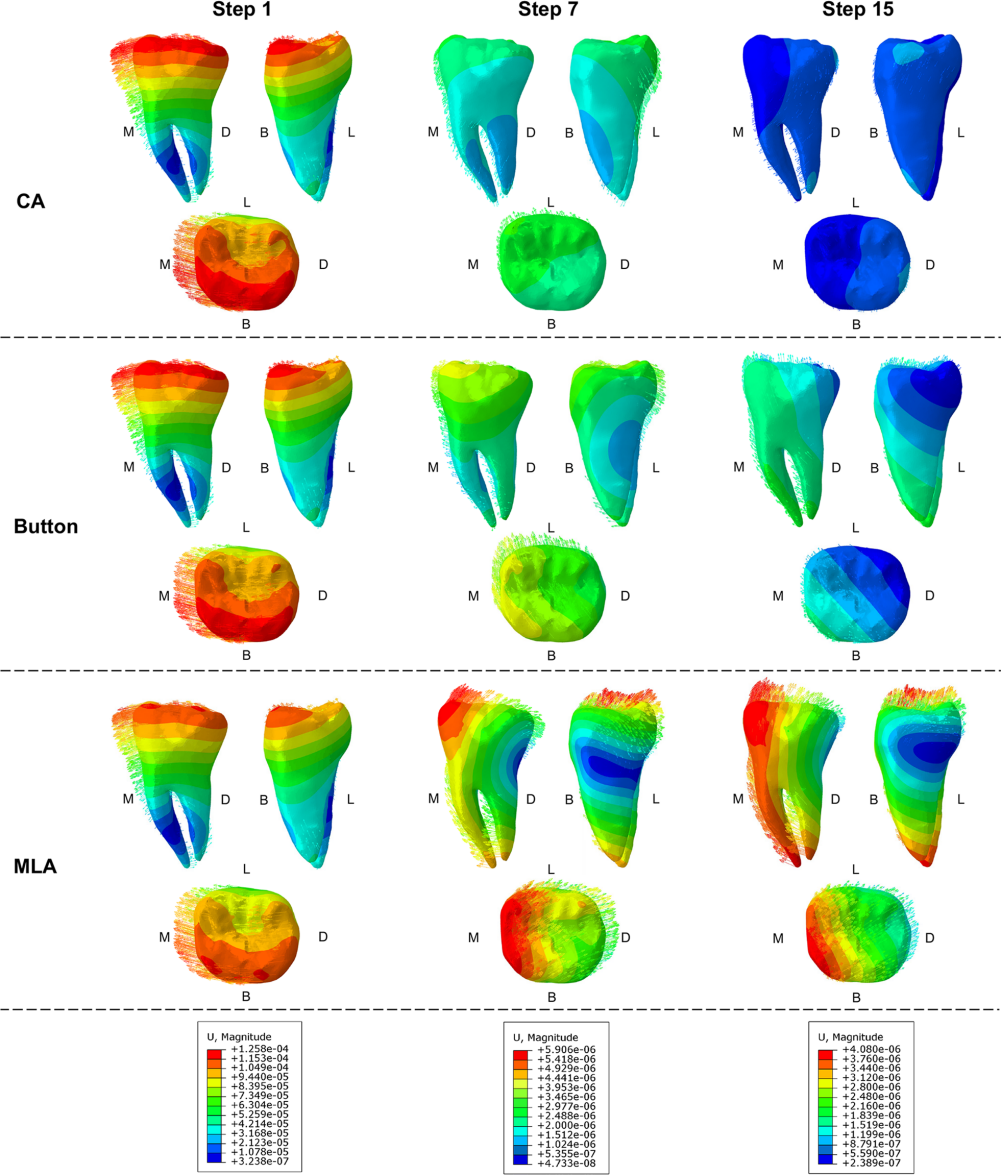
Displacement tendency of M2 at the beginning, intermediate, and final steps. *M, D, B, and L stand for mesial, distal, buccal, and lingual, respectively

Figure 7 showed the tooth displacement patterns at the beginning, intermediate, and final steps. In the 1st step, M2 tipped mesially in three groups, and the position of the rotation center was about one-third of the root length from the apex. In the 7th step, mesiolingual rotation was observed in CA and button groups, and the intrusion was greater in the CA group. On the contrary, the assistance of MLA caused the distal uprighting of M2. The rotation center was more occlusally displaced and distolingually centralized in the 15th than in the 7th step. In the 15th step, the rotation center was close to the mesial cusps in the CA group, but to the distolingual cusp in the button group. The root moved toward the mesial direction in both groups.

**Fig. 8:**
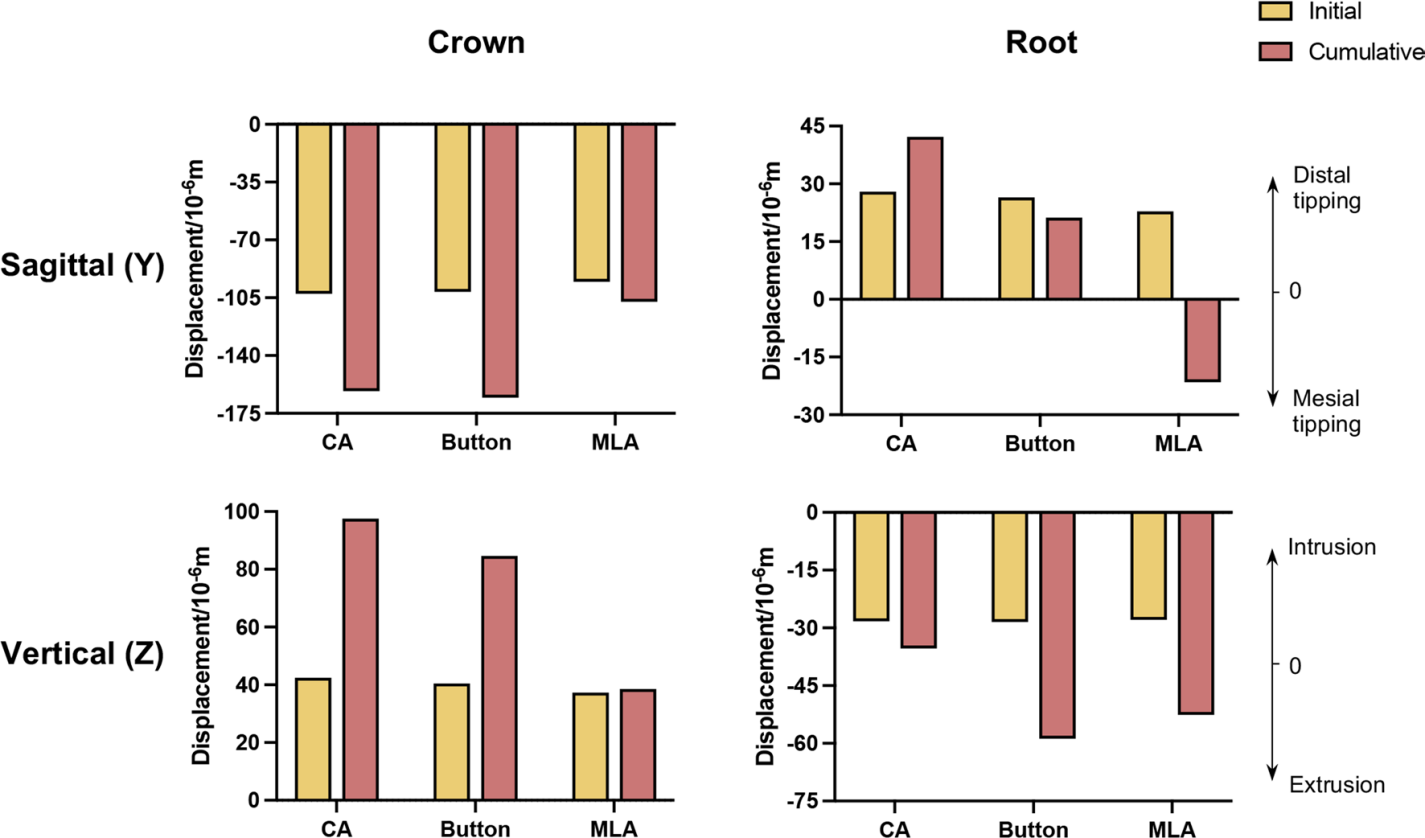
The comparison of the initial displacement and the cumulative displacement

The initial and cumulative displacement of the crown and root in *Y*- and *Z*-axes were presented in Fig. 8. The maximum crown displacement in *Y*-axis in the CA group increased from 0.1026 to 0.1651 mm, with a total increase of 36.5%. In the button and MLA group, the maximum displacement in the *Y*-axis of the M2 was also increased by 38.7% and 11.4%, respectively. Significant differences were observed in root displacement with the assistance of MLA in the *Y*-axis. The initial displacement was + 0.0228 mm, whereas the final displacement was − 0.0215 mm, with the opposite direction. In the *Z*-axis, the crown intrusion in the MLA group was the smallest (0.0386 mm), and the root extrusion in the CA group was the smallest ( − 0.0354 mm).

**Fig. 9:**
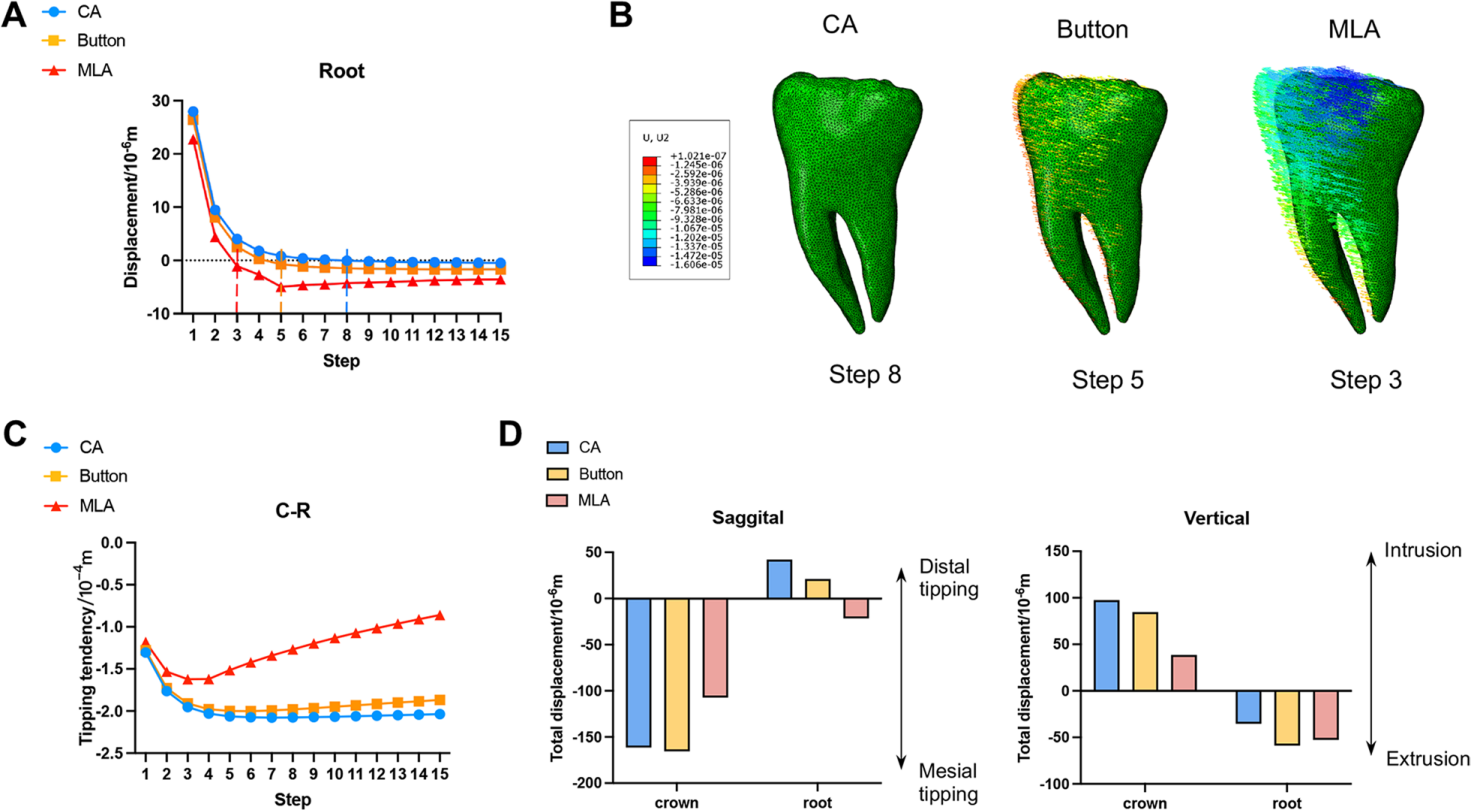
The long-term tooth movements.** A** The root movement at each iteration in the sagittal direction. **B** Tooth movement patterns in CA, button, and MLA groups at the 8th, 5th, and 3rd steps, respectively. **C** The curve of tipping tendency. **D** Cumulative displacement of crown and root in the sagittal and vertical direction

Figure 9 displayed the long-term tooth movements produced by CA with and without the button and MLA. In the sagittal direction, M2 inclined to the mesial, and the root moved to the distal at first (Fig. 9A). Later, the root gradually moved to the mesial. The roots started to move mesially in CA, button, and MLA groups at the 8th, 5th, and 3rd steps, respectively, and the movement is shown in Fig. 9B.

By calculating the displacement difference between the crown and the root (*C*–*R*) in each iteration, the mesial tipping curve of the tooth was depicted in Fig. 9C. The mesial inclination of M2 in the MLA group was apparently smaller than that of the other two groups, showing a trend of tipping first and gradually upright at the 5th step. The overall displacement values of both crown and root were negative only in the MLA group, proving the movement in the same direction (Fig. 9D). The crown and root of the CA and button groups moved in opposite directions. The mesial movement of the crown in the button group was greater than that in the CA group, and the distal movement of the root was smaller than that in the CA group.

**Fig. 10:**
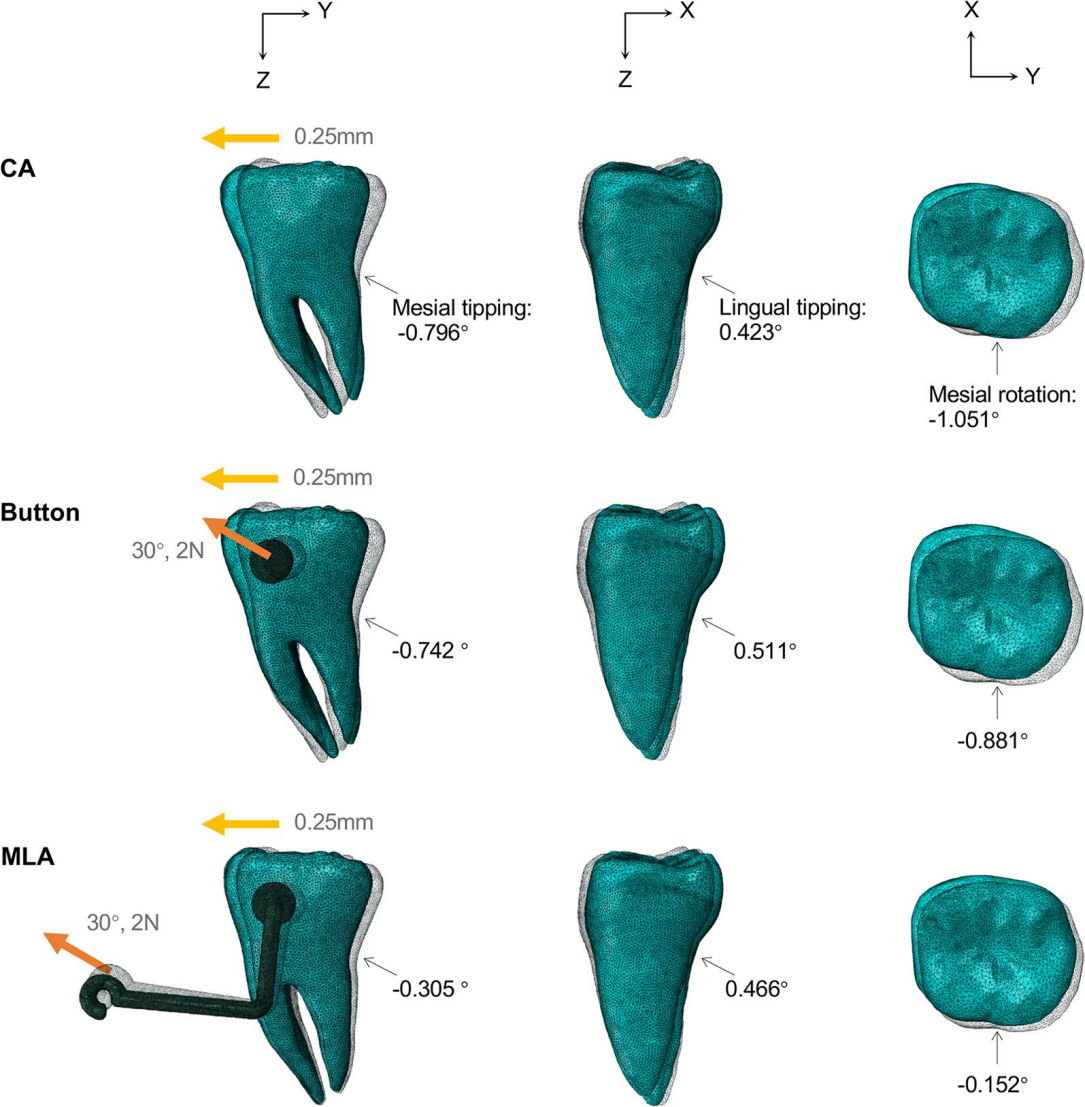
The cumulative rotation angle of M2 in three planes at the end of the iteration

As shown in Fig. 10, the angles of CA, button, and MLA group for mesial tipping were − 0.796°, − 0.742°, and − 0.305°, respectively; lingual tipping angles were 0.423°, 0.511°, and 0.466°, respectively; and mesial rotation angles were − 1.051°, − 0.881°, and − 0.152°, respectively.

## Discussion

The mechanical characteristic of CA and its long-term mechanical force changes remains unknown during the computer-aid design process, and the predictability of molars movement is not as expected. Hence, this work proposed an innovative iterative FE method to simulate the long-term tooth movement of CA and auxiliary devices and analyze the biomechanical changes of MM exerted by the dual-mechanical system.

Orthodontic tooth movement is not a one-step process. Our finding hinted that there was a significant difference between the initial displacement in the first step and the cumulative displacement at the end of the iteration, as depicted in Fig. 8. This difference was more evident in a dual-mechanical system with CA and auxiliary devices and less evident in a single mechanical system. Particularly, in the root movement along the Y-axis of the MLA group, the initial and final displacement direction was the opposite, which proved that it is unreliable if only the deformation of the first step is used as the predicted tooth movement.

The stress and movement amount decreased continuously in the CA group, while in the button and MLA groups, it reduced first and then stabilized within a certain range (Table 4). Moreover, in Fig. 4, the compressive stress of PDL was observed at the mesial cervix and distal apex in the first step, and the corresponding alveolar bone in this area would be absorbed [29]. Tensile stress was mainly concentrated on the distal cervix and mesial apex, and bone would be deposited in this area [29]. Therefore, M2 tipped mesially. As the iteration number increased, the stress distribution changed particularly in the button and MLA groups. A 2N force was loaded on the auxiliary devices, and the additional mechanical system increased tooth movement complexity, which has not been reported in the literature before. The concentration of compressive stress moved to the mesiobuccal side of PDL, and tensile stress moved to the distobuccal apex. Therefore, M2 tended to be upright gradually.

**Fig. 11:**
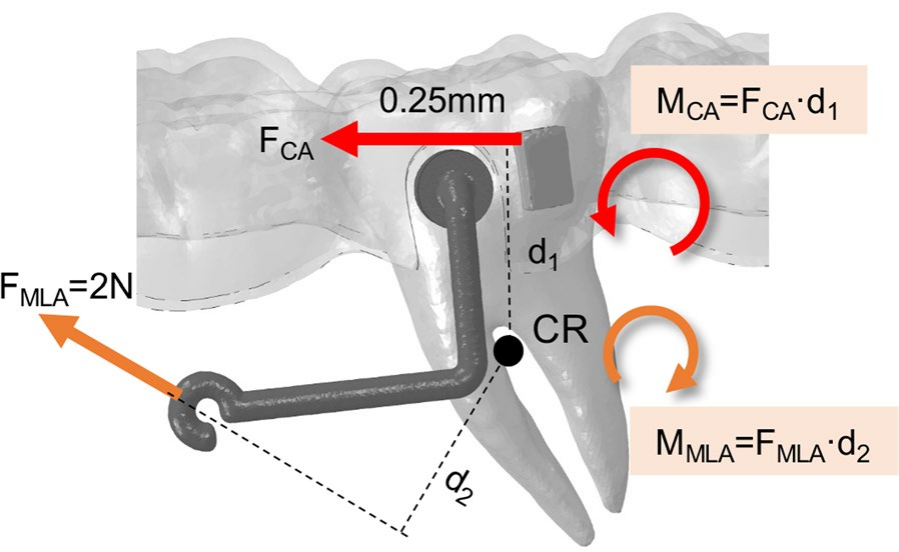
Mechanical systems acting on M2. The red arrows indicate the force delivered by CA, and the orange arrows indicate the force delivered by MLA

The CA alone caused undesigned mesial tipping, mesial rotation, and lingual tipping of M2. Similarly, a retrospective study found that molars achieved greater mesial tipping, mesial translation, and intrusion than predicted ﻿in extraction cases treated with Invisalign [30]. It was reported that the materials of CA are not as rigid enough as the archwire of traditional fixed appliances to retain the edentulous shape during closure [31–33], which might result in molar crown tipping. In addition, since the CA applied forces only on the exposed crown area, orthodontic force passed through the occlusal side of the center of resistance, leading to the inclination of teeth (Fig. 11). The mesial tipping and mesial rotation angle in the button group decreased by 0.054° and 0.17°, respectively, but the lingual tipping angle increased by 0.088°. It implied that the button commonly used in clinics is inefficient in relieving the mesial tipping of molars, and attention should be paid to avoid greater lingual inclination.

In order to upright and extrude M2, the MLA was innovatively designed by the principle of cantilever mechanics. By applying force to the mesial end of MLA, a molar uprighting moment was effectively produced, as shown in Fig. 11. Compared with the CA Group, although lingual tipping in the MLA group increased slightly by 0.043°, mesial tipping and rotation effectively decreased by 0.491° and 0.899°. It is in agreement with the results found by Barros et al. [34] that cantilevers were prone to cause distolingual molar rotation. The fulcrum positions of rotation of M2 were centralized more distally, lingually, and occlusally from the 1st to 15th steps (Fig. 7). In the vertical direction, the intrusion of the crown in the MLA group was smaller than that of the other two groups. Indeed, the combination of MLA applied an additional distal tipping moment and extrusive force on M2, which alleviated the side effects of mesial tipping and intrusion by CA alone. The proposed MLA has more effectiveness in promoting the bodily movement of molars than the traditional button, which is a promising therapeutic method for MM.

Under the action of the dual-mechanical system, the M2 tipped mesially first and then was upright through the mesial movement of the root. Therefore, tooth movement and force expression can be divided into two stages. In the first stage, the moment of aligner (*M*_CA_) was greater than the moment of MLA (*M*_MLA_), the aligner was the main mechanical system, and M2 tipped in a counterclockwise direction. As the tooth gradually moved toward the targeted position within the same aligner, the mismatch between the aligner and the teeth decreased, the deformation of the aligner decreased, and the force of the aligner (*F*_CA_) attenuated subsequently. Therefore, the stress and displacement were maximum at the beginning, but decreased exponentially, which was in agreement with the in vitro experiment of CA performed by Simon et al. [31] These findings demonstrated that the aligner exerted a constantly changing mechanical system on dentition, and the force changes according to the shape variable of the aligner material, which was in line with the previous study [35]. In the second stage, *M*_CA_ was smaller than *M*_MLA_, and the effects of continuous tractive force gradually dominated. M2 tended to tip in a clockwise direction, but due to the restriction of the aligner at the crown, it reflects in the mesial movement of the root. In general, the amount and direction of the crown and root movement altered as the mechanical system changed.

In addition, the stress of attachments and auxiliary devices is mainly concentrated on their interfaces with the tooth. These interface stresses may cause stress fracture and eventually destroy the integrity. The selection of orthodontic bonding cement should be carefully considered, and the interface should be handled carefully to avoid debonding of attachments, the button, and MLA.

This study demonstrated that the iterative FE method can simulate the long-term tooth movement patterns and related biomechanics under the complex mechanical system with continuous changes, laying a foundation for subsequent improvements in prediction accuracy and CAT efficiency. However, it still suffers from some limitations. The iterative FE models represent dynamic movement with cumulative initial displacement, which cannot fully simulate the actual clinical tooth movement process. The effect of MLA promoting MM should be validated clinically. Moreover, the biological response might differ from different tooth morphology, alveolar bone volume, and aligner material properties, which should be taken into consideration in future studies. More biological experiments in vitro and in vivo related to orthodontic bone remodeling are expected to provide data for establishing relevant mathematical models.

## Conclusions

- The force delivered by CA decreased exponentially during tooth movement. The aligner was the main mechanical system at first, and then, the additional system exerted by the button and MLA dominated gradually.
- M2 achieved greater mesiolingual tipping and mesial rotation than planned. The additional force system produced by MLA provided an effective distal tipping and extrusive moment. The MLA showed more benefits in reducing undesigned mesial tipping and rotation of M2 compared to the traditional buttons, which provided a therapeutic method for MM in patients using CAT.
- The direction and magnitude of tooth movement changed during the iterations. Since there was a significant difference between the initial displacement and the total long-term tooth movement, it is unreliable to take the first step displacement as the predicted movement trend.
- The proposed iterative FE method integrated in vitro mechanical experiment ﻿is an effective tool for simulating and predicting tooth movement under the guidance of the mechanical characteristic of CA and its long-term mechanical force changes.

## Data Availability

The datasets used and/or analyzed during the current study are available from the corresponding author on reasonable request.

## Abbreviations

CA: Clear aligner
CAT: Clear aligner therapy
MM: Molar mesialization
MLA: Modified lever arm
PDL: Periodontal ligament
M2: The second molar
FEA: Finite element analysis
CBCT: Cone beam computed tomography
